# UTP11 promotes the growth of hepatocellular carcinoma by enhancing the mRNA stability of Oct4

**DOI:** 10.1186/s12885-023-11794-2

**Published:** 2024-01-17

**Authors:** Yan Chen, Xiaowei Zhang, Mingcheng Zhang, Wenting Fan, Yueyue Lin, Guodong Li

**Affiliations:** 1 Department of Gastroenterology, The Second Affiliated Hospital of Shandong First Medical University, Tai an City, China; 2 Department of Endoscopy Center, The Second Affiliated Hospital of Shandong First Medical University, Tai an City, China; 3 Department of Gastrointestinal Surgery, The Second Affiliated Hospital of Shandong First Medical University, 271000 Tai an City, China

**Keywords:** UTP11, Tumor stemness, Oct4, mRNA stability, Hepatocellular carcinoma

## Abstract

**Background:**

Several publications suggest that UTP11 may be a promising gene engaged for involvement of hepatocellular carcinoma (HCC) pathology. However, there are extremely limited biological, mechanistic and clinical studies of UTP11 in HCC.

**Methods:**

To anayze the UTP11 mRNA expression in HCC and normal clinical samples and further investigate the correlation between UTP11 expression and pathology and clinical prognosis via the Cancer Tissue Gene Atlas (TCGA) database. The protein levels of UTP11 were checked using the Human Protein Atlas (HPA) database. GO-KEGG enrichment was performed from Cancer Cell Line Encyclopedia (CCLE) database and TCGA dataset. The levels of UTP11 were tested with qRT-PCR and western blotting assays. Cell viability, immunofluorescence and flow cytometry assays and animal models were used to explore the potential involvement of UTP11 in regulating HCC growth in vitro and in vivo. The correlation of UTP11 and tumor stemness scores and stemness-associated proteins from TCGA database. The mRNA stability was treated with Actinomycin D, followed by testing the mRNA expression using qRT-PCR assay.

**Results:**

UTP11 was highly expressed in HCC samples compared to normal tissues from TCGA database. Similarly, UTP11 protein expression levels were obviously elevated in HCC tissue samples from HPA database. Furthermore, UTP11 levels were correlated with poor prognosis in HCC patient samples in TCGA dataset. In addition, the UTP11 mRNA levels was notably enhanced in different HCC cell lines than in normal liver cells and knocking down UTP11 was obviously reduced the viability and cell death of HCC cells. UTP11 knockdown suppressed the tumor growth of HCC in vivo experiment and extended the mice survival time. GO-KEEG analysis from CCLE and TCGA database suggested that UTP11 might involve in RNA splicing and the stability of mRNA. Further, UTP11 was positively correlated with tumor stemness scores and stemness-associated proteins from TCGA database. Knockdown of UTP11 was reduced the expression of stem cell-related genes and regulated the mRNA stability of Oct4.

**Conclusions:**

UTP11 is potentially a diagnostic molecule and a therapeutic candidate for treatment of HCC.

**Supplementary Information:**

The online version contains supplementary material available at 10.1186/s12885-023-11794-2.

## Background

HCC constitutes the fourth most prevalent contributor to carcinoma-related fatalities globally [1, 2]. Despite extensive studies on HCC in recent years that have led to an increasing understanding of its biology, a large proportion of drivers remain incurable. Multigenerational kinase inhibitors, such as levatinib and sorafenib, as first-line therapies for patients with HCC are currently the most common treatments [3]. Although some efficacy has been achieved with the use of immunotherapies (e.g. nivolumab), however, their response rates are very low (less than 20%) [4]. Under this situation, it is urgently requested new prognostic biomarkers or treatment strategies to be implemented to enhance the prognosis of patients with HCC.

Cancer stem cells (CSCs) represent a critical subset of tumor cells, sharing functional characteristics with normal stem cells [5]. Despite their limited numbers, they possess attributes such as self-renewal, unrestricted proliferation, and multidirectional differentiation, enabling evasion of immune surveillance and playing a crucial role in tumor development [6]. The presence of CSCs accounts for various clinical phenomena in cancer, including HCC, such as nearly inevitable tumor recurrence post successful chemotherapy or radiotherapy, tumor dormancy, and development of therapeutic resistance [7]. Hence, precise targeting of CSCs holds potential to enhance the therapeutic effectiveness against cancer. Promising therapeutic strategies are actively being developed, focusing on targeting CSCs, specifically in HCC [8, 9].

Understanding the molecular mechanisms underlying the behavior of CSCs in specific cancers, such as HCC, is crucial for developing effective therapeutic interventions. Identifying key genes and proteins that regulate CSCs can provide valuable insights into potential targets for tailored therapies. U3 small nucleolar RNA-associated protein 11 (UTP11) is one such protein of interest encoded by the *UTP11* gene [10, 11]. UTP11, in conjunction with the small subunit (SSU) procesome, plays a vital role as a component of the U3-snoRNA-containing complex, participating in the processing of small subunits of the eukaryotic ribosome [12]. Existing literature suggests that UTP11 may be a promising gene implicated in HCC pathology and could potentially serve as a predictive prognostic biomarker for HCC patients [13, 14]. However, biological, mechanistic and clinical studies of UTP11 in HCC are extremely limited. In this study, we have confirmed through analyses of the TCGA database and a series of in vitro and in vivo experiments that UTP11 indeed plays a role in promoting the growth of HCC. Furthermore, our exploration of its potential regulatory mechanism has revealed that UTP11 contributes to the stabilization of CSCs by enhancing the stability of CSC-related genes, including Oct4.

## Materials and methods

### TCGA database analysis

Download and assort RNAseq data from TCGA database (https://portal.gdc.cancer.gov) for HCC (TCGA-LIHC) and extract the data in transcripts per million reads (TPM) format for the normal as well as cancer samples. The RNAseq data in TPM format was then log2 transformed. The patient’s clinical information was downloaded from UCSC XENA (https://xenabrowser.net/datapages/) [15]. For the mRNA expression of UTP11, ggplot2 package (3.3.6) in R language (4.2.1) visualizes UTP11 mRNA levels from normal and LIHC clinical samples. ROC curves were obtained using the pROC package (1.18.0) and time-dependent ROC curves were analyzed via timeROC package (0.4). The survivals of UTP11 were tested using the survival package (3.3.1) for proportional risk hypothesis testing and fitted survival regressions, and the results were visualized using the survminer package. For GO and KEGG analysis, the pearson correlation test was first performed using cor.test to obtain with significantly different genes, then performed with the clusterProfiler R package [16–18]. The relevance of UTP11 to other genes was analyzed by correlation analysis, and the analysis results were visualized by co-expression heat map using ggplot package. Tumor stem cell characteristics were derived from the transcriptome data of LIHC samples obtained from TCGA [19]. The association between the expression levels of UTP11 and scores indicating tumor stemness was subjected to statistical analysis using the Spearman test.

### Validation of protein levels of UTP11 in HPA database

UTP11 protein levels were verified by immunohistochemical (IHC) assay in normal and hepatocellular carcinoma tissues, and the data were obtained from the HPA database (https://www.proteinatlas.org/), which is an IHC-based database for protein expression analysis [20–22].

### CCLE database analysis

The CCLE database was used to analyze UTP11 expression levels at the cellular levels for GO and KEGG analysis. The CCLE database (https://sites.broadinstitute.org/ccle/) contains genomic data for more than 1100 cell lines from various types of cancers, including HepG2, Huh7, SK-Hep-1, SNU-182, and a total of 25 HCC cell lines [23]. Data were mainly obtained by high-throughput sequencing, containing copy number, mRNA expression (Affymetrix), reverse-phase protein array, RNA sequencing and reduced representative bisulfite sequencing. UTP11-related differential genes were identified by pearson analysis, and heat maps were derived by utilizing the pheatmap R package (1.0.12). Differential genes were ID-transformed by org.Hs.eg.db package, GO-KEGG enrichment was carried out by clusterProfiler package [16–18]. Adjustment of P value is performed by BH method.

### Cell transfection

Culture the cells in a 6-well plate overnight until they reach around 50% confluence on the day of infection. Replace the culture medium in each well with a 5 µg/ml Polybrene (Santa Cruz Biotechnology, sc-134,220) and medium mixture. Introduce UTP11 shRNA lentiviral particles (Santa Cruz Biotechnology, sc-88,082-V) into the culture medium to infect the cells, gently swirling the plate for even distribution, and then incubate overnight. Approximately 8 to 12 h later, the complete medium was replenished. Subsequently, choose stable shRNA-expressing clones through puromycin selection (2 to 10 µg/ml).

### Real-time quantitative PCR (RT-qPCR)

TRIzol reagent (Invitrogen) was applied to extraction of total RNA from HCC cells. 500 ng of RNA was reversely transcribed into cDNA (Takara Biotechnology), and then mRNA expression was detected by SYBR Green method (Takara Biotechnology). The sequences of the primers used in this study were as follows: UTP11 F: TCGGAAGAAGGCTCTTGAAA, R: GCTTCTGCAACCCTCTTCAT; Oct4 F: 5′-CTTGAATCCCGAATGGAAAGGG-3′, R: 5′-GTGTATATCCCAGGGTGATCCTC-3′; Nanog F: 5′-ACCTATGCCTGTGATTTGTGG-3′, R: 5′-AGTGGGTTGTTTGCCTTTGG-3′; Sox2 F: 5′-GCCGAGTGGAAACTTTTGTCG-3’, R: 5’-GGCAGCGTGTACTTATCCTTCT-3’; CD133 F: 5′-AGTCGGAAACTGGCAGATAGC-3′, R: 5′-GGTAGTGTTGTACTGGGCCAAT-3′; GAPDH F: 5’-TGACTTCAACAGCGACCCA-3’, R: 5’-ACCCTGTTGCTGTAGCCAAA-3’. The statistical approaches of RT-qPCR were conducted with the 2^−ΔΔCt^ method.

### Western blotting assay

HCC cells were collected, lysed using RIPA buffer, and the concentration of protein was testing using the BCA kit (ThermoFisher Scientific). Total protein (30 µg per sample) was separated within an SDS PAGE gel at 80 V for 2 h and transferred to a PVDF membrane (Millipore) at 4 °C for 2–3 h. Membranes were treated with 5% milk (diluted in PBS-T solution (1×PBS containing 0.1% Tween-20), follwed by incubating with primary antibodies (UTP11, abcam, #ab247068, 1:1000 dilution; Oct4, abcam, ab18976; GAPDH, Santa Cruz, #sc-47,724, 1:1000 dilution) overnight at 4 °C. After washing with PBS-T buffer, the membranes were incubated with secondary antibody (Cell Signaling Technology, #14,708 or #14,709, 1:10,000 dilution) for 2 h at room temperature. Blots were processed using ECL reagent (ThermoFisher Scientific).

### Cell proliferation assay

To measure the cell viability of HCC cells, the Cell Counting Kit-8 (CCK-8) reagent (Beyotime Biotechnology, #C0037) was applied. Briefly, HCC cells (2000 cells /well) were seeded in a 96-well plate, and after adding CCK-8 solution (10 µl per well), incubation was continued for 1 h in a cell incubator, and absorbance was measured at 450 nm.

For EdU (5-ethynyl-2’ -deoxyuridine) assay, HCC cells were seeded in confocal Petri dishes. To be tested, incubate cells with medium containing 10µM EdU for 3 h at 37 °C. After washing with 1×PBS for three times, the HCC cells were followed by fixing with 4% paraformaldehyde (PFA) and blocking with permeability solution (1×PBS containing with 0.3% Triton X-100). Then cells were stained with reaction solution (Beyotime Biotechnology, #C0075S).

### Flow cytometry analysis

To detect the cell death status, cells were collected and washed with 1×PBS. After resuspending with 500 µl of PBS, the HCC cells were further incubated with 5 µM 7-AAD (invitrogen) at 37˚C for 30 min, followed by flow cytometric analysis.

### Sphere formation assay

HCC cells were cultured at a density of 1000 cells/well. The serum-free medium utilized for cultivating spheres consisted of DMEM/F12 medium supplemented with epidermal growth factor (EGF, 20 ng/ml), fibroblast growth factor (FGF, 20 ng/ml), 1% GlutaMax, 1% nonessential amino acids, 2% B27 supplement.

### Mice experiment

For the xenograft model, the 6-8-week-old male nude mice were subcutaneously injected with 1 × 10^6^ control or UTP11 knockdown HCC-LM3 cells on the back of the mice (5 mice per group). The sizes of the subcutaneous tumors were measured 1–2 times per week and the mice were euthanized after four weeks. For the orthotopic model, the 6–8 weeks male nude mice were inoculated with 2 × 10^5^ control or UTP11 knockdown HCC-LM3 cells (5 mice per group) in the left lobe of the mouse liver. All mice were raised under pathogen-free conditions. Tumor size was measured 1–2 times per week using ultrasound, and the volume based on ultrasound measurement was calculated as V = (length × width^2^)/2. Mice were euthanized when they showed weight loss of more than 20% or decreased activity. Animal experiments were approved by the animal ethics of the Second Affiliated Hospital of Shandong First Medical University (approval number 2023-002).

### Immunofluorescence assay

Tissue samples were fixed with 4% PFA overnight at 4 °C and then treated with 30% sucrose solution until the tissue settled. Then tissue samples were embedded with OCT and cut into 10 μm sections. The slides were treated with acetone at -20 °C for 5 min and blocked with permeability solution (1×PBS containing with 0.3% Triton X-100), followed with blocking in 5% BSA at room temperature for 1 h. The slides were treated with primary antibody (Ki67, abcam, #ab16667, 1:250 dilution; PCNA, abcam, #ab92552, 1:200 dilution) overnight at 4 °C and the next day incubated with secondary antibody (Invitrogen, #A-31,572, 1:500 dilution; Invitrogen, #A-11,008, 1:500 dilution). Nuclei were stained by DAPI reagent (1:1000 dilution). The slides were observed and imaged using a confocal microscope.

### Statistical analysis

All statistical analyses were performed using R language 4.2.1 and GraphPad Prism 7.0 software. Two-tailed t-test was used for two groups analysis. Survival curves were performed using the Kaplan-Meier method. *P* < 0.05 was considered to be statistically significant.

## Results

### UTP11 expression was higher in HCC samples compared to normal tissues

**Fig. 1:**
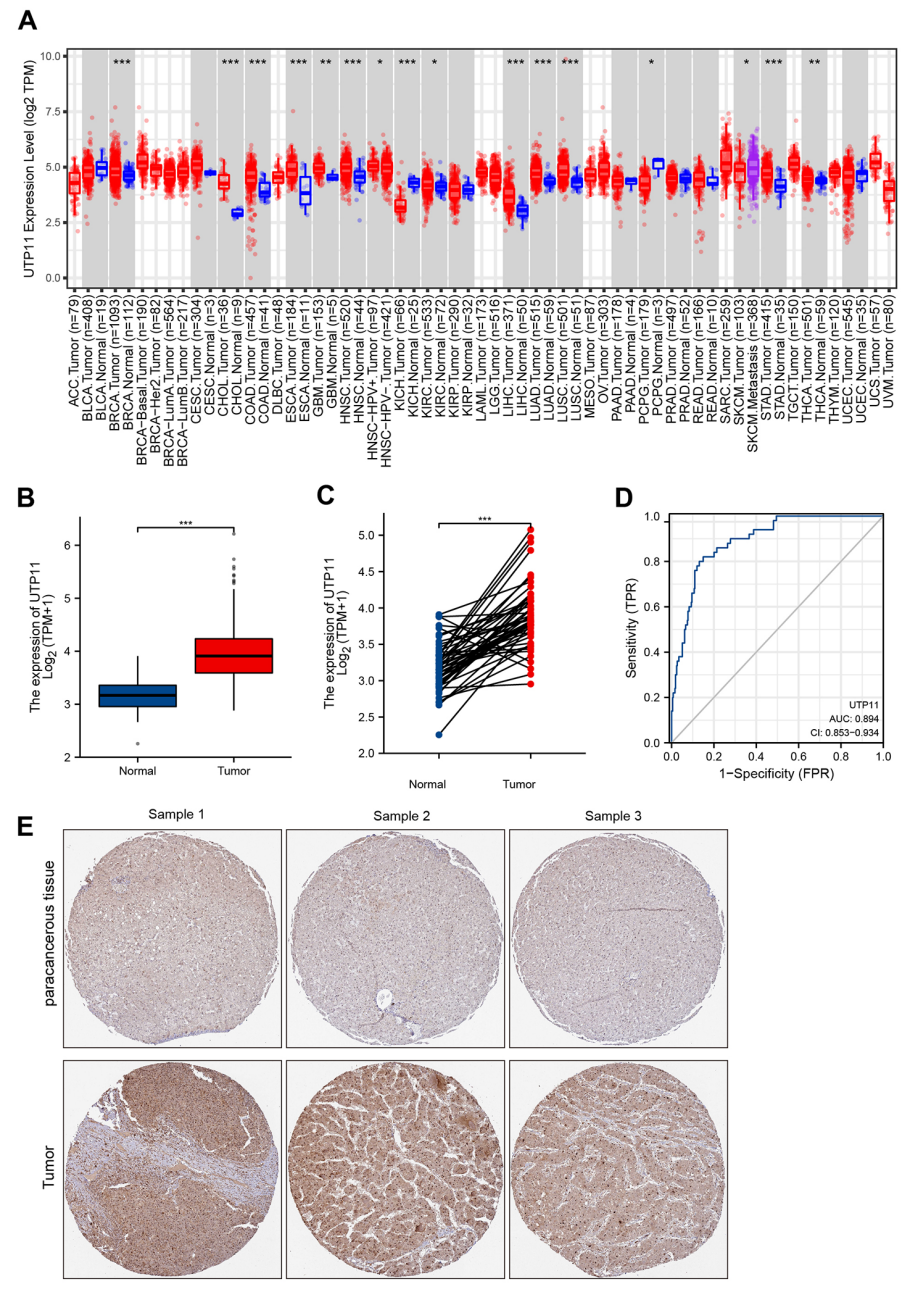
UTP11 expression was higher in HCC samples compared to normal tissues. (**A**) UTP11 expression levels in 33 tumor tissues compared to normal tissues from the TCGA database generated by TIMER2.0 database (http://timer.comp-genomics.org/) [44]. The expression of UTP11 was represented using box plots. (**B**) The expression levels of UTP11 in HCC and normal paracancerous tissues from TCGA database. (**C**) UTP11 was highly expressed in tumor tissues than in normal tissues in 50 pairs of HCC paired samples from TCGA database. (**D**) The ROC curve analysis of UTP11 was performed in predicting tumor versus normal outcome. The horizontal coordinate is the False Positive Rate (FPR) and the vertical coordinate is the True Positive Rate (TPR). (**E**) The protein expression levels of UTP11 in paracancerous and HCC tissues were detected by IHC method which was generated by HPA database. * *P* < 0.05; ** *P* < 0.01; *** *P* < 0.001

We first investigated the UTP11 mRNA expression levels from TCGA database of an extensive number of tumors in comparison with adjacent normal tissues, and the data displayed that UTP11 showed high levels in most tumors compared to normal tissues, including hepatocellular carcinoma tissues (Fig. 1A and B). In addition, UTP11 was remarkably observed higher in HCC clinical samples than in normal samples in 50 pairs of HCC paired samples (Fig. 1C). Early diagnostic challenges and the deficiency of specific diagnostic molecules are among the primary causes of the very poor survival rate of HCC patients [24, 25]. The results of the ROC curve to predict HCC and normal tissue outcomes indicated that the area under the curve (AUC) reached 0.894 and that UTP11 expression levels were able to accurately predict whether HCC or normal tissue was present (Fig. 1D). Next, we examined the UTP11 protein expression in HCC and normal clinical samples by the HPA database based on the IHC method to detect protein expression. Similar to the UTP11 mRNA levels in the TCGA database, UTP11 was obviously enhanced in HCC samples compared to normal ones (Fig. 1E).

### UTP11 expression correlates with poor prognosis in HCC patient samples

**Fig. 2:**
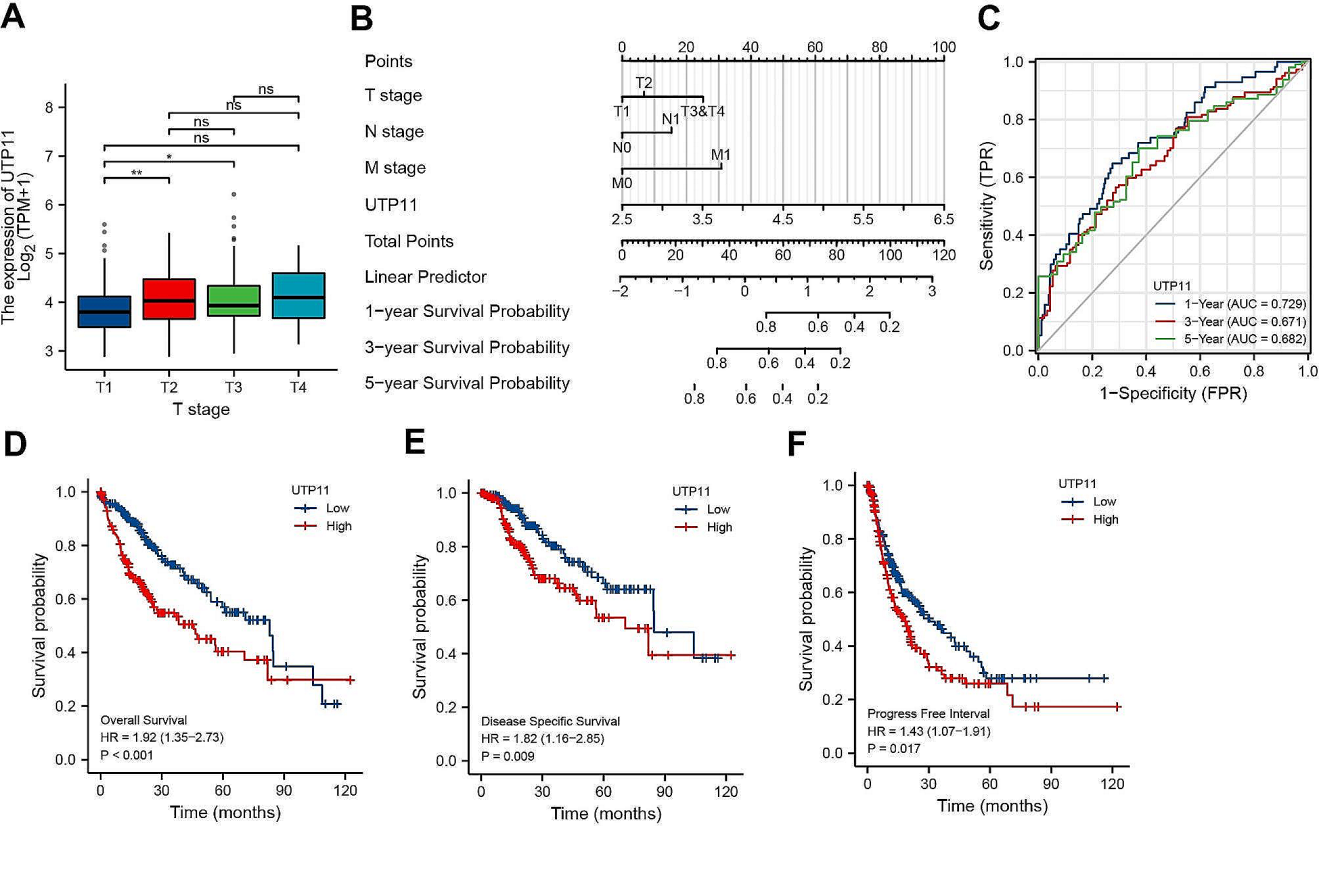
UTP11 expression correlates with poor prognosis in HCC patient samples. (**A**) The expression levels of UTP11 in different patient T stages from TCGA database. (**B**) Nomogram diagram showing the correlation between clinical stages and UTP11 expression levels of HCC patients and patient prognosis from TCGA database. (**C**) The time-dependent ROC curves showed the correlation between UTP11 and HCC prognosis from TCGA database. (**D**-**F**) The correlation between UTP11 expression and overall survival (**D**), disease specific survival (**E**) and progress free survival (**F**) of HCC patients. * *P* < 0.05; ** *P* < 0.01; ns, no significance

To characterize the interaction of UTP11 with the pathologic features and prognosis of HCC patients, we first examined the UTP11 levels in different patient stages. Our obtained findings displayed that the UTP11 levels were markedly raised in high T stages (T2 and T3) than in low stage (T1), suggesting that UTP11 expression may be associated with worse clinical stages (Fig. 2A). Univariate and multifactorial analyses revealed that UTP11 can represent as a great predictor of independent prognosis in patients with HCC (Fig. 2B). The time-dependent ROC curves illustrated that the 1-, 3- and 5-year AUC areas for the correlation between UTP11 and HCC prognosis were 0.729, 0.671 and 0.682, respectively; indicating that UTP11 has a predictive effect on HCC prognosis (Fig. 2C). In addition, UTP11 levels demonstrated a strong correlation with the prognosis of HCC patients, with high expression of UTP11 showing significantly worse prognosis (Fig. 2D and F).

### UTP11 promotes the growth of liver cancer cells in vitro

**Fig. 3:**
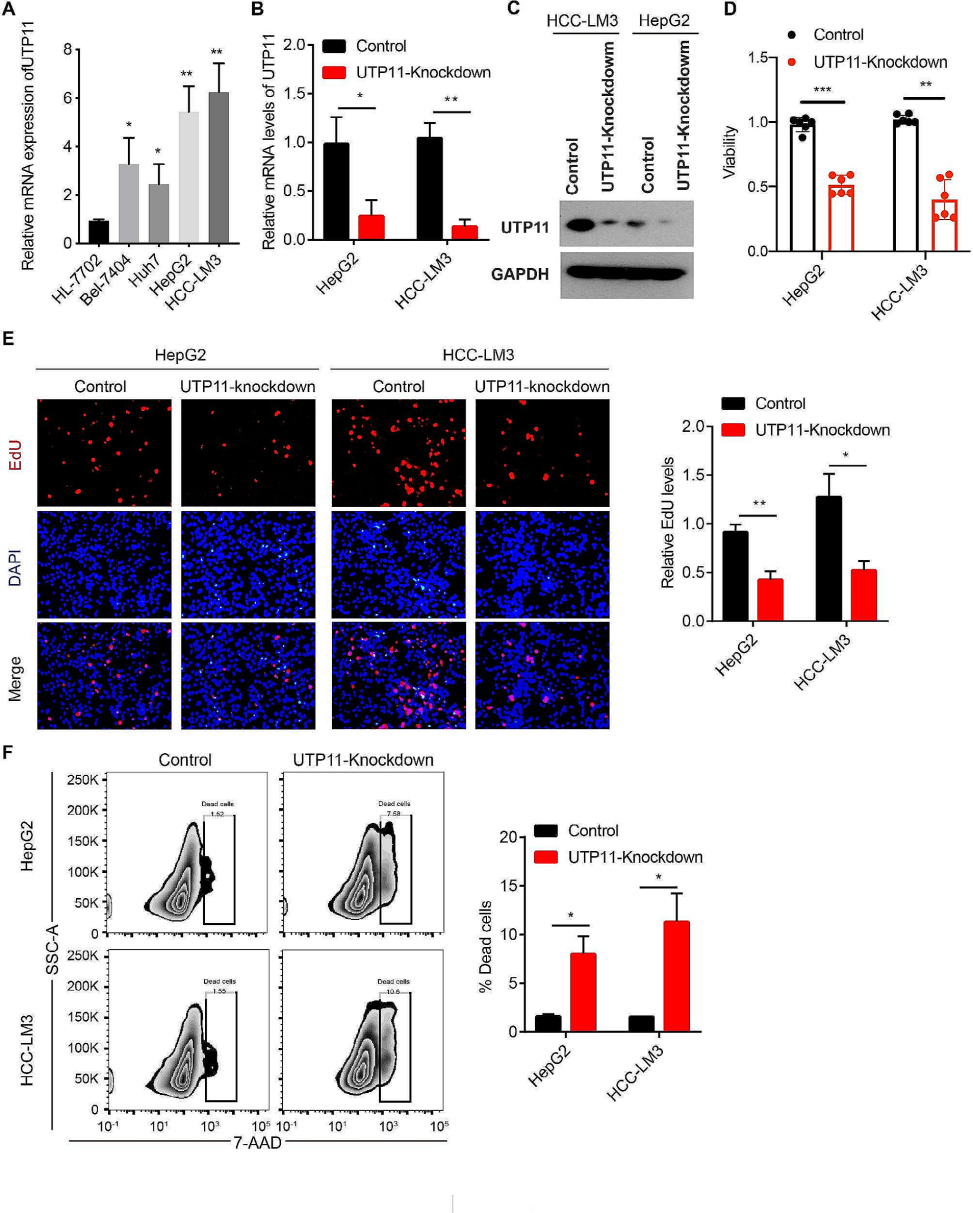
UTP11 promotes the growth of liver cancer cells in vitro. (**A**) The mRNA expression levels of UTP11 in normal human liver cell line (HL-7702) and liver cancer cell lines (Bel-7404, Huh7, HepG2, HCC-LM3) were detected by qRT-PCR assay. (**B**) The mRNA levels of UTP11 in control or UTP11-knockdown HepG2 and HCC-LM3 cells were examined by qRT-PCR assay. (**C**) The protein expression of UTP11 in control or UTP11-knockdown HepG2 and HCC-LM3 cells were tested using western blotting assay. (**D**) The viability of control or UTP11-knockdown HepG2 and HCC-LM3 cells was checked using CCK8 assay. (**E**) The proliferation was detected using EdU staining in control or UTP11-knockdown cells. (**F**) HepG2 and HCC-LM3 cells treated with shUTP11 for 7 days (UTP11 knockdown group), followed with 7-AAD staining and detected by flow cytometry. * *P* < 0.05; ** *P* < 0.01; *** *P* < 0.001

As a further verification of the utilization of UTP11 on liver cancer, we firstly inspected the UTP11 levels in normal human liver cells (HL-7702) and HCC cell lines (Bel-7404, Huh7, HepG2, HCC-LM3) by qRT-PCR assay. The levels of UTP11 were remarkably enhanced in liver cancer cell lines compared to HL-7702 cells, with the highest expression levels in HepG2 and HCC-LM3 cell lines (Fig. 3A). Therefore, we knocked down the UTP11 levels in HepG2 and HCC-LM3 cells and verified the knockdown efficiency by qPT-PCR and western blotting assays, the findings presented that the UTP11 levels were greatly decreased in the UTP11 knockdown group compared to controls (Fig. 3B and C). To detect the impact of UTP11 on the cell growth of HCC cells, our CCK8 data revealed that the cell viability of HepG2 and HCC-LM3 cells in the UTP11 knockdown group at day 7 was significantly reduced compared to controls (Fig. 3D). Similarly, immunofluorescence results of EdU staining showed a strong reduction in the proliferation rate of HepG2 and HCC-LM3 cells in response to UTP11 knockdown (Fig. 3E). Furthermore, HepG2 and HCC-LM3 cells treated with shUTP11 for 7 days (UTP11 knockdown group) showed a higher rate of dead cells compared to the controls by flow cytometry assay (Fig. 3F).

### UTP11 knockdown inhibits the growth of liver cancer in vivo

**Fig. 4:**
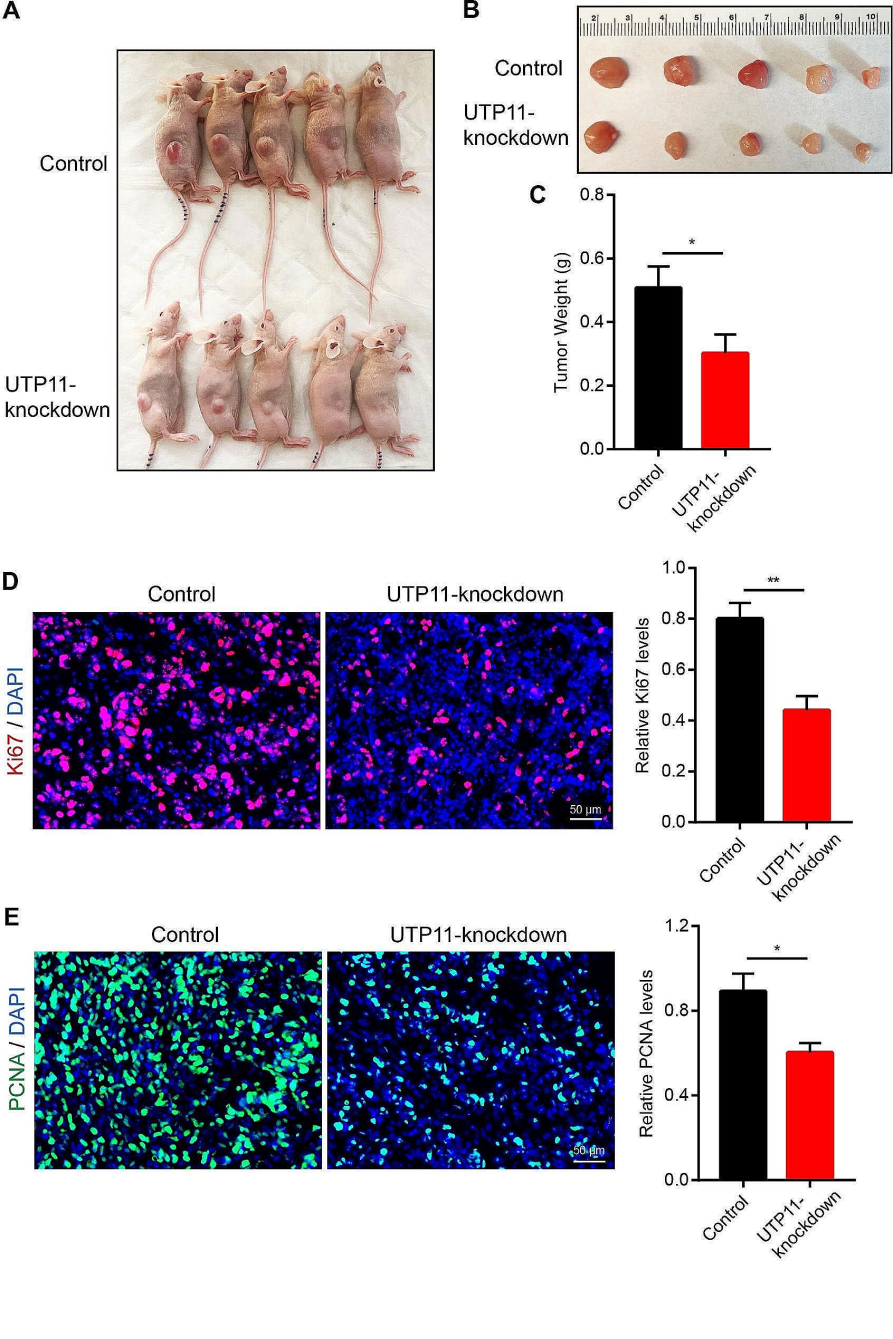
UTP11 knockdown inhibits the growth of liver cancer of xenograft model. (**A**-**C**) Control or UTP11 knockdown HCC-LM3 cells were injected into the back of nude mice, the tumor sizes and weights of HCC-LM3 cells was tested. (**D**, **E**) The Ki67 and PCNA expression was detected by immunofluorescence assay in control or UTP11-knockdown tissues. * *P* < 0.05; ** *P* < 0.01

**Fig. 5:**
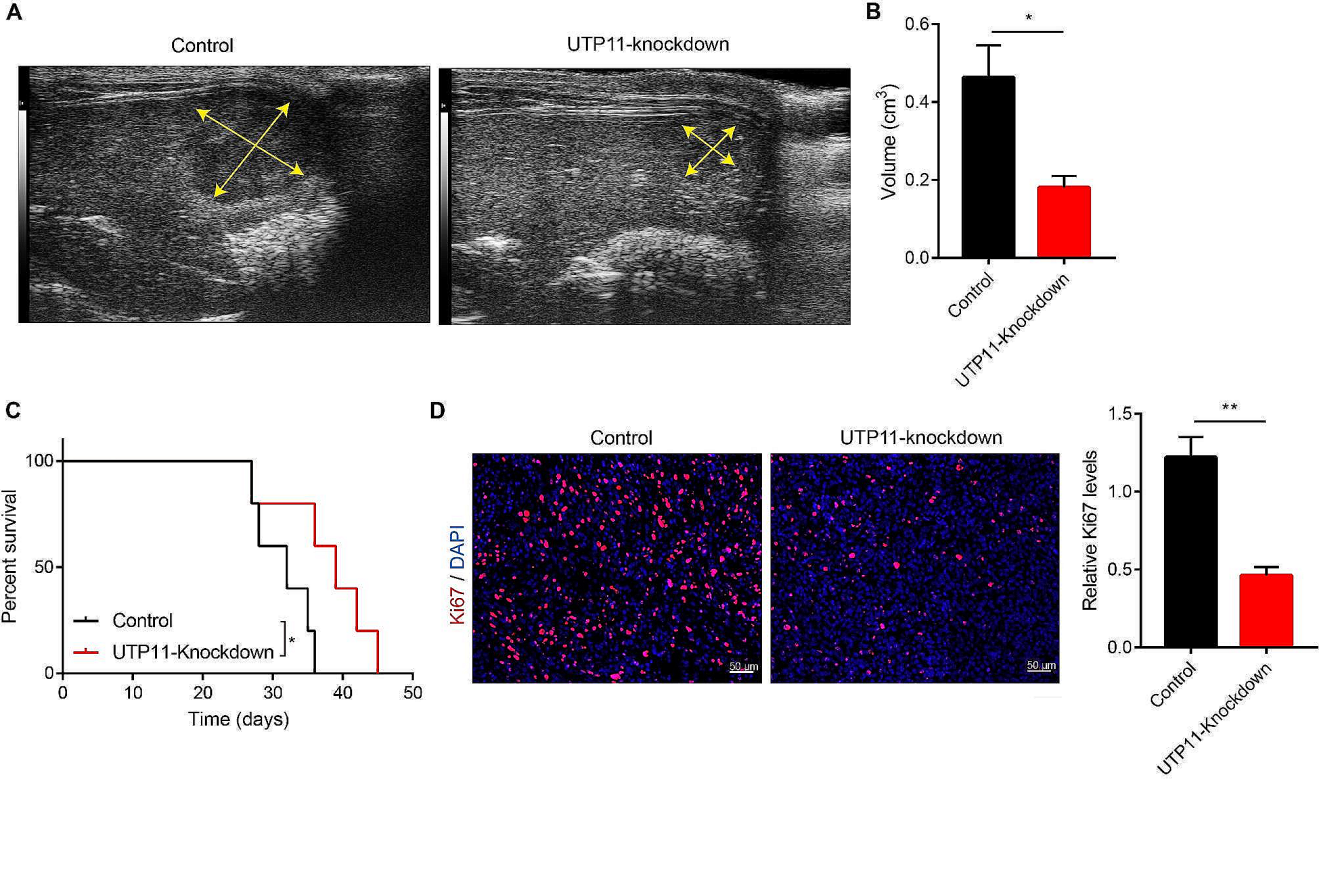
UTP11 knockdown inhibits the growth of liver cancer of orthotopic model. (**A**) Control or UTP11 knockdown HCC-LM3 cells were injected into the liver of nude mice, the growth of HCC-LM3 cells was examined by ultrasound. (**B**) The volume of liver cancer in the control or UTP11 knockout group was detected by ultrasound. (**C**) Survival experiment was performed with the control or UTP11 knockout group (*n* = 5 per group). (**D**) The Ki67 expression was detected by immunofluorescence assay in control or UTP11-knockdown tissues. * *P* < 0.05; ** *P* < 0.01

Next, to examine the impact of UTP11 expression levels on the growth of HCC in vivo. We first used xenograft model to detect the effect of UTP11 knockdown on subcutaneous tumor growth. The findings demonstrated that the tumors in the knockdown group of UTP11 were significantly smaller than controls (Fig. 4A and C). Additionally, the protein levels of Ki67 and PCNA, the proliferating indicators of cancer, were found to be greatly reduced in the UTP11 knockdown samples (Fig. 4D and E). Next, we injected 1 × 10^6^ of control or UTP11 knocking down HCC-LM3 cells into the liver of nude mice and examined the growth of HCC by ultrasound. Similarly, our ultrasound data indicated that the sizes of liver cancers in the UTP11 knocking down groups were clearly shrinking to those in the controls (Fig. 5A and B). The survival data displayed that the UTP11 knockdown group was substantially extended (Fig. 5C). Moreover, Ki67 and PCNA levels were showed to be obviously decreased in the UTP11 knockdown samples (Fig. 5D). These data indicated that UTP11 could promote the growth of liver cancer in vitro and in vivo.

### UTP11 may be involved in the regulation of RNA splicing and the stability of mRNA

**Fig. 6:**
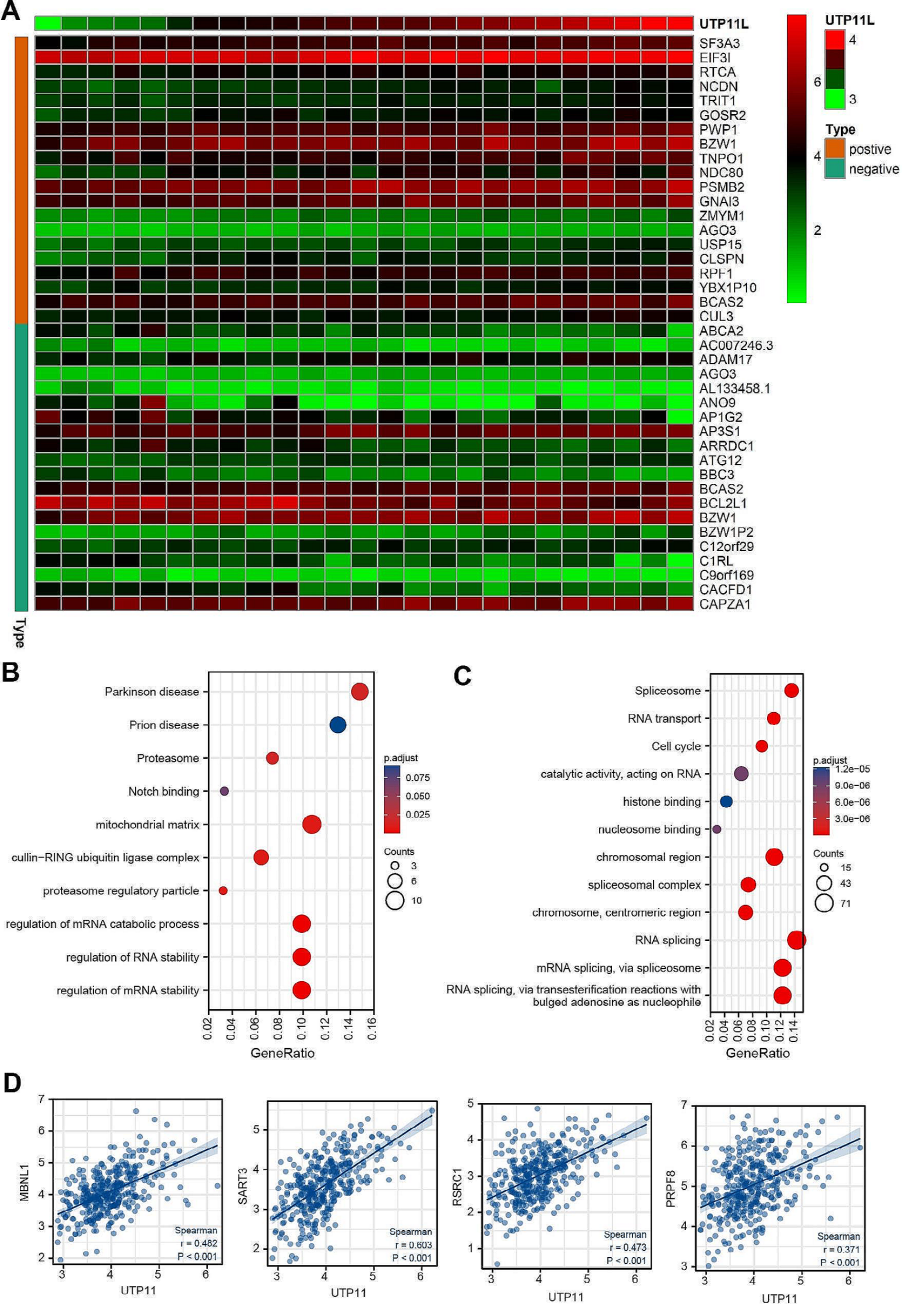
UTP11 may be involved in the regulation of RNA splicing and the stability of mRNA. (**A**) The heat map showed the top 20 genes that were positively or negatively associated with UTP11 gene expression from the CCLE database. (**B**) GO and KEEG analysis were used to examine the functional and signaling pathways associated with UTP11 expression in HCC cell lines from the CCLE database. (**C**) GO and KEEG analysis were used to examine the functional and signaling pathways associated with UTP11 expression in HCC cell lines from TCGA database. (**D**) The correlation of UTP11 expression and RNA splicing-related proteins MBNL1, SART3, RSRC1 and PRPF8

With a view to gaining further knowledge on the mechanism of how UTP11 promotes the growth of liver cancer, we first used the CCLE database, a database containing 25 HCC cell lines, to test the functions of UTP11 involved in liver cancer cells. We displayed a heat map of genes positively or negatively associated with UTP11 gene expression in the CCLE database (Fig. 6A). Next, we carried out GO-KEEG functional and enrichment pathway analysis, and the data suggested that UTP11 expression was linked to proteasome regulation, mRNA stability regulation, and regulation of the mRNA catabolic process (Fig. 6B).

Moreover, we analyzed the function of UTP11 expression levels from TCGA database by GO-KEEG. Interestingly, the expression of UTP11 was related to RNA splicing, which involved in regulating the mRNA stability (Fig. 6C). In addition, UTP11 was positively correlated with RNA splicing-related proteins MBNL1, SART3, RSRC1 and PRPF8 (Fig. 6D). Taken together, UTP11 may be responsible for RNA splicing and mRNA stability.

### UTP11 may mediate tumor stem cells in HCC by stabilizing the mRNA of OCT4

**Fig. 7:**
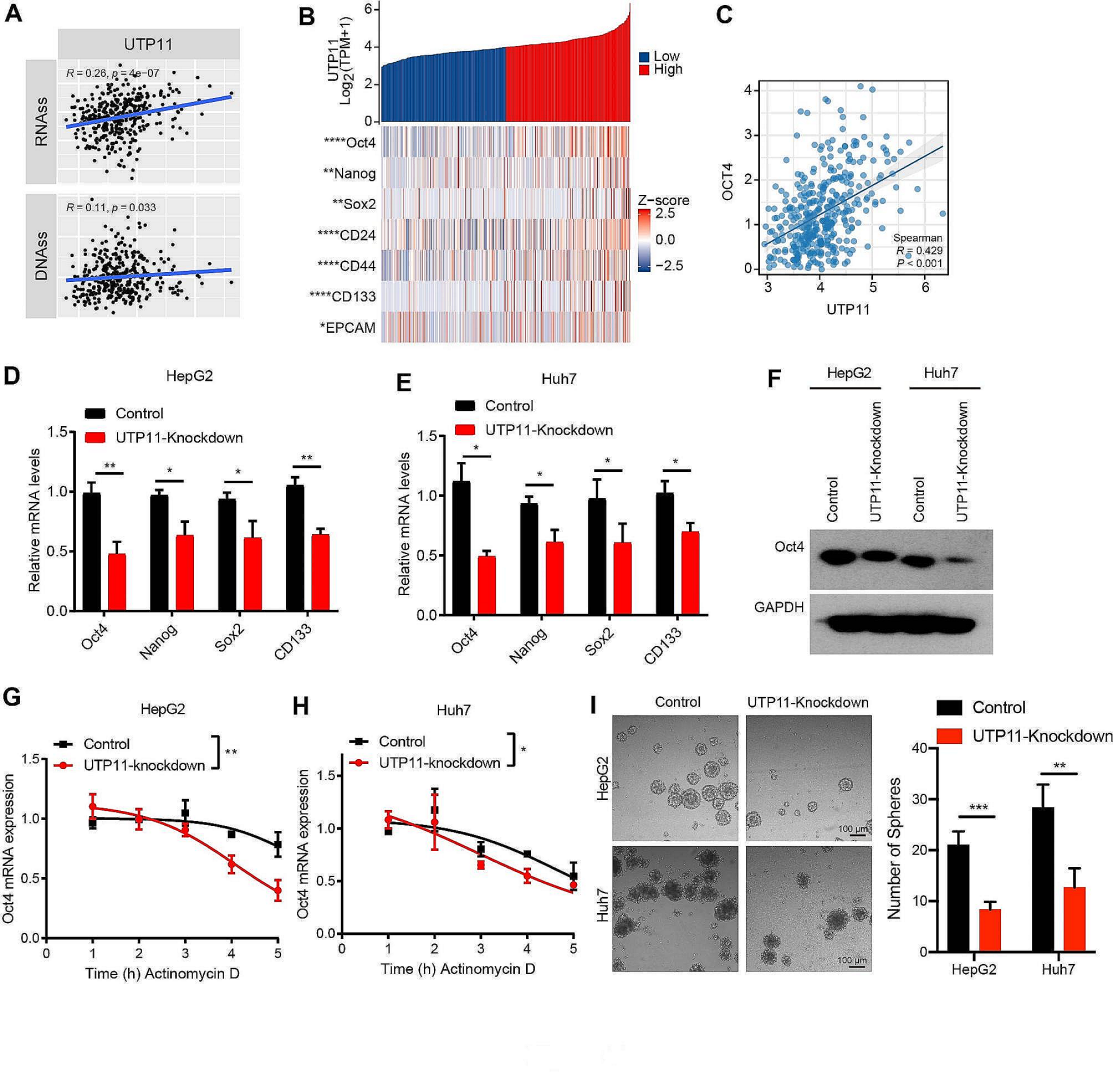
UTP11 may mediate tumor stem cells in HCC by stabilizing the mRNA of OCT4. (**A**) The correlation between UTP11 expression and tumor stemness scores from TCGA database. (**B**) Heat map showed the correlation between UTP11 expression and stemness-associated proteins (Oct4, Nanog, Sox2, CD24, CD44, CD133, EPCAM) from TCGA database. (**C**) The association between UTP11 and Oct4 expression from TCGA database. (**D**, **E**) The mRNA levels of stem cell-related genes, including Oct4, Nanog, Sox2, and CD133, were detected in control or UTP11 knockdown of HepG2 and Huh7 cells by qPR-PCR assay. (**F**) The change in Oct4 expression in the absence of UTP11 was detected at the protein level by western blot assay in HepG2 and Huh7 cells. (**G**, **H**) The control or UTP11 knockdown of HepG2 and Huh7 cells was treated with 5 µg/ml of Actinomycin D, followed by testing the mRNA expression of Oct4 using qRT-PCR assay. (**I**) HepG2 and Huh7 cells with or without UTP11 were subjected to sphere formation analysis. * *P* < 0.05; ** *P* < 0.01; *** *P* < 0.001; **** *P* < 0.0001

To validate the involvement of UTP11 in tumor stemness in liver cancer, we analyzed with TCGA database to detect the relationship between tumor stemness scores and mRNA levels of UTP11. The results pointed out that the expression of UTP11 was linked to RNA and DNA stemness scores, which implied that with higher expression levels of UTP11, the tumor stemness was stronger (Fig. 7A). Next, we analyzed the relevance of UTP11 expression to tumor stemness-associated proteins through heat map, which showed a positive correlation between UTP11 expression and tumor stemness-associated proteins (Fig. 7B), with the strongest correlation with OCT4 (Fig. 7C).

It has been well-documented that the expression of Oct4 is notably low in HCC-LM3 [26]. Given that HepG2 and Huh7 cells exhibit characteristics resembling liver stem cells [27, 28], we opted to use HepG2 and Huh7 cells as our targeted cell lines. We conducted qRT-PCR to examine various remaining stem cell-related factors, including Oct4, Nanog, Sox2, and CD133, the mRNA levels of stem-related factors were significantly reduced after knockdown of UTP11 both in HepG2 and Huh7 cell lines (Fig. 7D and E). Notably, the most pronounced decrease in expression was observed for Oct4. Similarly, the protein expression level of Oct4 was significantly decreased after UTP11 knockdown by western blot assay (Fig. 7F). As our results in Fig. 5 revealed that UTP11 might be involved in mRNA stability, we hypothesized whether UTP11 downregulated the expression of Oct4 by regulating the mRNA stability of OCT4. Therefore, we detected the mRNA levels of Oct4 by treating control or UTP11 knockdown cells with Actinomycin D (5 µg/ml), followed by testing with qRT-PCR assay. The data demonstrated that UTP11 increased the stability of OCT4, with UTP11 knockdown causing de-stabilization of OCT4 (Fig. 7G and H). Additionally, the downregulation of UTP11 led to a significant suppression in the generation of sphere formation, suggesting that UTP11 plays a crucial role in promoting the formation of these specialized cell spheres (Fig. 7I).

## Discussion

The survival rate of HCC is dramatically low attributed to the difficulties of early diagnosis, rapidly progressive disease and deficiency of controlled drugs [25, 29]. The presence of aberrant heterogeneity greatly limits the progress of early HCC studies and the detection of living cancers [25]. Hence, the identification of a prevalent molecular regulator in HCC that can distinguish clearly between tumor tissues and paraneoplastic tissues to obtain an early diagnosis. Several reports have suggested that UTP11 may be a promising gene involved in HCC pathology and may be a prognostic predictive biomarker for HCC patients [13, 14]. Our data indicated that UTP11 levels were much higher in HCC samples compared to normal tissues; ROC curves revealed that UTP11 was able to discriminate considerably between liver cancer tissues and paraneoplastic samples, which are consistent with the previously reported literatures. Further, UTP11 expression in HCC patient samples was linked to nfavorable survival outcomes. However, previous reports on the function of UTP11 in HCC are very limited, where our results demonstrated the capability of UTP11 to promote the growth and proliferation of HCC in vitro and in vivo.

RNA splicing is an important biological process in the processing and maturation of tRNA, rRNA, especially mRNA, and is one of the critical molecular diversity mechanisms for generating proteins [10, 11]. UTP11 is a small nucleolar RNA-associated protein, and we showed that UTP11 is involved in the RNA splicing process by exploring the results of GO-KEGG analysis of TCGA database. The RNA splicing process is very complex, there are plenty of factors involved in it. SART3 (also known as hPrp24) is a central spliceosomal component, a unique spliceosomal protein that selectively binding to U6 and is involved in U6 biogenesis, function in splicing, and recycling [30–32]. PRPF8, as the core of a scaffolding protein stabilizing spliceosome, whose rearranged conformation benefits the three-dimensional encapsulation of the catalysing active of U2/U6 RNA, is one of the largest and most conserved protein, directly involved in splicing fidelity [33, 34]. MBNL proteins serve as suppressors or activators of RNA splicing in a variety of transcripts [35]. RSRC1, previously known as SRrp53, is localized to the nuclear speck where it binds to the U2 small ribonucleoprotein (snRNP) cofactor (U2AF35), which has roles in both constitutive and selective precursor mRNA splicing [36]. In this study, UTP11 was positively correlated with RNA splicing-related proteins MBNL1, SART3, RSRC1 and PRPF8, which further demonstrated that UTP11 may be involved in the RNA splicing process.

CSCs represent one of a small population of carcinoma cells capable of influencing self-renewal, differentiation and tumorigenesis [37]. The markers of HCC stem cell subsets have been identified, including EpCAM, CD24, CD133, CD44, Oct4, and Nanog [38–40]. CSCs are critical contributors to the recurrence, metastasis and chemoresistance of HCC [41]. Therefore, increasing studies are searching for treatments that target CSCs in an attempt to control tumor recurrence and metastasis [9, 42]. Our study showed that UTP11 may mediate tumor stem cells in HCC by stabilizing the mRNA of Oct4. These results provide insight into the potential of UTP11 as a therapeutic candidate for targeting HCC tumor stem cells. However, more experiments are needed to verify this speculation.

Certain modifications of RNA, such as pseudouridine (Ψ), N6,2’-O-dimethyladenosine (m6Am) and N6-methyladenosine (m6A) have proved to modulate the stability of mRNA and thus affect different cellular and biological processes [43]. Our data suggested that UTP11 expression was associated with proteasome regulation, mRNA stability regulation, and regulation of the mRNA catabolic process. However, whether UTP11 can mediate the mRNA stability of OCT4 by regulating the RNA modifications of Oct4 is unclear. This study also did not delve deeply into the specific mechanisms through which UTP11 regulates stem cells to promote hepatocellular carcinoma growth. In future research, we aim to thoroughly investigate and confirm the role of UTP11 in promoting hepatocellular carcinoma by regulating stem cells at both the cellular and animal levels, enhancing our understanding of this crucial mechanism.

In conclusion, UTP11 knockdown suppressed the tumor growth of HCC and extended the mice survival time. Mechanically, UTP11 might promote the regulation of the mRNA stability of Oct4. UTP11 is potentially a diagnostic molecule and a therapeutic candidate for treatment of HCC. By targeting UTP11, it may be possible to inhibit HCC growth and extend the survival time of patients. In addition, our findings offer novel insights into the molecular mechanisms that drive the progression of HCC, with a particular emphasis on the regulatory role of UTP11 in Oct4 expression.

### Electronic supplementary material

Below is the link to the electronic supplementary material.

Supplementary Material 1

## Data Availability

The datasets generated and/or analysed during the current study are available in TCGA, CCLE and TISCH database. The datasets that were used and/ or analyzed in this study are available upon request from the corresponding author.

## Abbreviations

HCC: Hepatocellular carcinoma
TCGA: The Cancer Tissue Gene Atlas
CCLE: Cancer Cell Line Encyclopedia
CSCs: Cancer stem cells
SSU: Small subunit
TPM: Transcripts per million reads
IHC: Immunohistochemical
RT-qPCR: Real-time quantitative PCR
CCK-8: Cell Counting Kit-8
EdU: 5-ethynyl-2’ -deoxyuridine
PFA: Paraformaldehyde
AUC: Area under the curve
snRNP: Small ribonucleoprotein
