# Supplementary figures and images for: UTP11 promotes the growth of hepatocellular carcinoma by enhancing the mRNA stability of Oct4
Source: BMC Cancer. 2024 Jan 17;24:93. doi: 10.1186/s12885-023-11794-2 (PMC10795422; doi:10.1186/s12885-023-11794-2)

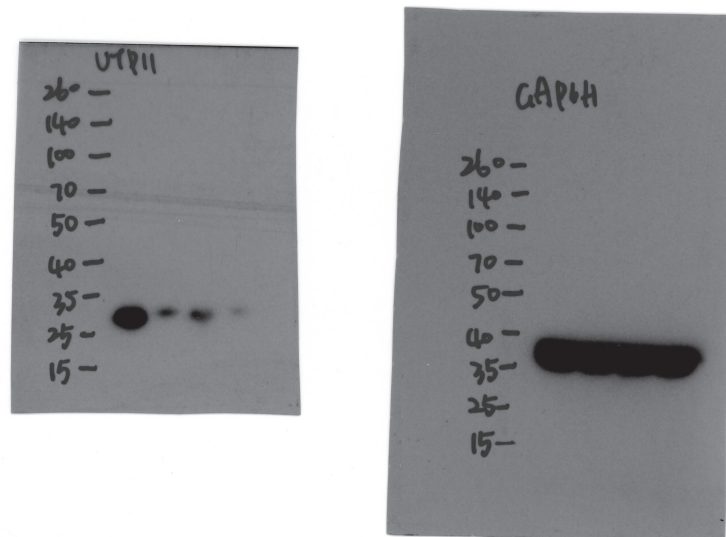

Figure 3C

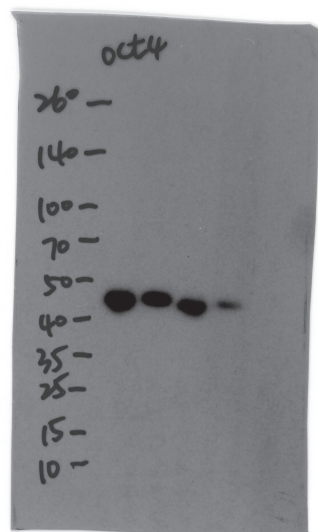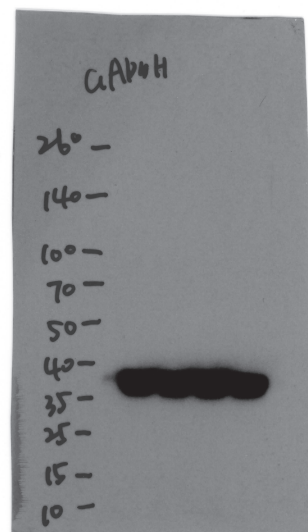

Figure 7F

Supplement: Supplementary file 1 — Supplementary Material 1 [file 12885_2023_11794_MOESM1_ESM.pdf]
